# Acute Hepatitis and Liver Failure Following the Use of a Dietary Supplement Intended for Weight Loss or Muscle Building — May–October 2013

**Published:** 2013-10-11

**Authors:** Sarah Y. Park, Melissa Viray, David Johnston, Ethel Taylor, Arthur Chang, Colleen Martin, Joshua G. Schier, Lauren S. Lewis, Kara M Levri, Kevin Chatham-Stephens

**Affiliations:** Hawaii Dept of Health; Div of Environmental Hazards and Health Effects, National Center for Environmental Health; EIS Officers, CDC

On September 9, 2013, the Hawaii Department of Health (HDOH) was notified of seven patients with severe acute hepatitis and fulminant liver failure of unknown etiology. Patients were previously healthy and sought medical care during May–September 2013. Clinicians reported that the seven patients had all used OxyELITE Pro, a dietary supplement marketed for weight loss and muscle gain, before illness onset.

The HDOH, with the CDC and the Food and Drug Administration (FDA), initiated a public health investigation including patient interviews, medical chart reviews, and collection of supplement samples for analysis. Subsequently, a case was defined as acute hepatitis of unknown etiology occurring on or after April 1, 2013 in a person who had consumed a weight loss or muscle-building dietary supplement within the previous 60 days and had a serum alanine aminotransferase level greater than or equal to four times the upper limit of normal (>160 IU/L) and a total bilirubin level greater than or equal to two times the upper limit of normal (>2.5 mg/dL) and a negative evaluation for infections including viral hepatitis. Excluded were other etiologies such as pre-existing autoimmune hepatitis, chronic alcohol use, and chronic liver diseases such as primary biliary cirrhosis, primary sclerosing cholangitis, Wilson’s disease, and hemochromatosis.

Clinicians reported 45 possible cases to the Hawaii DOH in response to a public health alert. Of those, 29 have been identified as cases. The patients have a median age of 33 years (range: 16–66); 14 (48%) were male. The date of first reported laboratory test was used as a proxy for illness onset and ranged from May 10 through October 3, 2013 (Figure). The most commonly reported symptoms included loss of appetite, light-colored stools, dark urine, and jaundice. Median laboratory values reported at the peak of illness were: aspartate aminotransferase 1,128 IU/L (range: 104–2,184, upper limit of normal ~40); alanine transaminase 1,793 IU/L (range: 347–3,091, upper limit of normal ~40); alkaline phosphatase 150 IU/L (range: 68–251, upper limit of normal ~120); and total bilirubin 12.6 mg/dL (range: 2.8–39.6, upper limit of normal ~1.2). Ten patients had liver biopsy data available at the time of this report; seven had histology consistent with hepatitis from drug/toxic injury, with findings including hepatocellular necrosis and cholestasis. Eleven (38%) patients were hospitalized, with a median duration of 7 days (range: 1–45). One patient died, two patients received liver transplants, and two remain hospitalized; all other hospitalized patients have been discharged.

Of the 29 identified patients, 24 (83%) reported using OxyELITE Pro during the 60 days before illness onset. Twelve (41%) reported use of OxyELITE Pro and no other dietary supplement, and 12 (41%) reported use of both OxyELITE Pro and at least one additional dietary supplement. Three (10%) reported using other dietary supplements for weight loss or muscle building, but not OxyELITE Pro. Information about use of OxyELITE Pro is not yet known for two (7%) patients. For twelve patients with specified dates of use, the median duration from starting OxyELITE Pro to the onset of symptoms was 60 days (range: 7–130). There was no other dietary supplement or medication use reported in common by more than two patients.

National case finding efforts have included surveillance of poison center data using the National Poison Data System. A call for cases was also disseminated through the United Network for Organ Sharing listserv to transplant programs across the country. These activities have identified four persons in states outside of Hawaii with reported OxyELITE Pro or other weight loss or muscle-building dietary supplement use prior to the development of acute hepatitis of unknown cause. One of these is a resident of Hawaii who obtained their product in Hawaii but was diagnosed in a different state. CDC, in collaboration with state health departments, is collecting additional clinical and epidemiologic information from these persons to determine if this outbreak is nationwide.

Results from FDA product testing are pending. While the investigation is ongoing and these data are preliminary, clinical data, laboratory tests, and histopathology of liver biopsy specimens collected thus far suggest drug- or herb-induced hepatotoxicity. Drug- and herb-induced hepatotoxicity have been reported in association with exposure to a variety of drugs and herbs used as dietary supplements and can lead to severe acute hepatitis and liver failure (1,2). Drug- and herb-induced hepatotoxicity often resolves following discontinuation of the product (3). Attributing liver injury to a specific ingredient can be challenging because of multiple ingredients, product variability, and lack of testing to confirm exposure to a product. Clinicians evaluating patients with acute hepatitis should ask about consumption of dietary supplements as part of a comprehensive evaluation. Clinicians should report patients meeting the case definition to the local or state health department, as well as the FDA’s MedWatch program. Clinicians can discuss patient management options with a medical or clinical toxicologist by calling their local poison center at 1-800-222-1222. Persons who use dietary supplements for weight loss or muscle gain should do so with caution and under a medical provider’s close supervision.

## Figures and Tables

**FIGURE:**
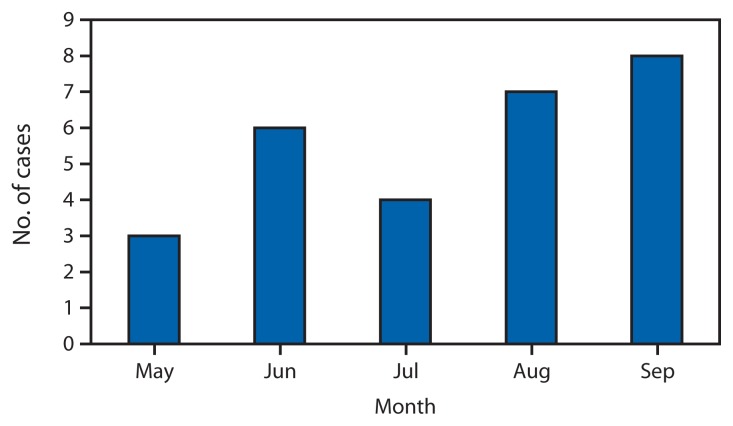
Number of cases by date of first reported laboratory result* * Three cases with a first reported laboratory result in October are not shown.
